# Biomonitoring of urban ultrafine particle exposure and associated health effects

**DOI:** 10.3389/ijph.2026.1609855

**Published:** 2026-09-01

**Authors:** Elizabeth Miriam Fireman, Einat Fireman Klein, Mor Dabach, Emilia Hardak

**Affiliations:** 1 Department Environmental Occupational Health, Gray Faculty Medical Health Science, Tel Aviv, Israel; 2 Pulmonary Division Carmel Medical Center, Rappaport Faculty of Sciences, Technion Institute Technology, Haifa, Israel; 3 Pulmonary Department, Ichilov Hospital Tel Aviv University, Tel Aviv, Israel; 4 Pulmonary Unit, Bnai Zion Medical Center Rappaport Faculty of Sciences, Technion Institute Technology, Haifa, Israel

**Keywords:** human, inflammation, bio-monitoring and evaluation, ultra-fine particles (UFP), urban areas

## Abstract

**Objective:**

To assess the health impact of industrial and urban air pollution in the Haifa Bay area using biological monitoring of micro- and ultrafine particles (UFPs), and to examine their associations with functional, inflammatory, and clinical outcomes.

**Methods:**

The study included 99 hospital staff members: 50 from Bnai Zion Medical Center in the Haifa Bay area and 49 from Tel Aviv Medical Center as controls. Participants underwent spirometry, fractional exhaled nitric oxide (FeNO) measurement, exhaled breath condensate (EBC) and saliva collection, and completed a health questionnaire. Assessments were repeated after 1 year. Latent class analysis (LCA) was used to identify homogeneous subgroups based on biological and clinical parameters.

**Results:**

LCA identified a cluster characterized by higher body mass index, increased UFP concentrations in EBC, and elevated salivary LDH-calcium levels. This cluster consisted predominantly of participants from Haifa and showed higher salivary lymphocyte counts. Participants in this subgroup also reported more frequent respiratory symptoms, including cough, sputum production, and atopic complaints.

**Conclusion:**

Biological monitoring of nanosized particles may provide early indicators of adverse health effects associated with urban-industrial air pollution exposure.

## Introduction

Epidemiologic studies have demonstrated a clear association between exposure to airborne particles at concentrations commonly found in major metropolitan areas worldwide and increased mortality rates [1, 2]. Ambient airborne particulate matter (PM) is ubiquitous; however, its concentrations, particle sizes, and chemical compositions can vary substantially across locations and over time [3]. The aerodynamic diameter is defined as the diameter of a spherical particle with a density of 1 g/cm^3^ [4]. PM with an aerodynamic diameter of less than 10 µm (PM10) is further classified into size-based categories: coarse particles (2.5–10 µm), fine particles (PM2.5, <2.5 µm), and ultrafine particles (UFP or PM0.1, <0.1 µm or 100 nm).

Most studies have relied on data obtained from environmental monitoring, which provides valuable information for assessing regulatory compliance in pollution control but is a poor surrogate for measuring the full extent of particulate matter’s adverse effects on the human respiratory system. To better understand these impacts, it is important to consider measurements of a pollutant’s internal dose, which account for the route of exposure (e.g., inhalation, dermal absorption) and variations in the delivered dose at the individual level [5]. The internal dose of pollutants can be accurately assessed only through biological monitoring. In this context it was shown that exposure to metal (loid)s was evaluated by measuring the levels of aluminum, arsenic, cadmium, chromium, mercury, and nickel in the urine of 100 participants from urban residential areas in Iran [6]. Tear fluid has also been reported as a useful matrix for biomonitoring environmental and chemical exposures [7]. Biomonitoring of particulate matter (PM) and polycyclic aromatic hydrocarbons (PAHs) was conducted among children attending schools in polluted urban and industrial areas. These children, exposed to higher levels of PM and PAHs, exhibited increased concentrations of urinary PAH metabolites compared to children from rural areas [8].

Assessing particulate burden in the lungs can be performed using a combination of fiberoptic bronchoscopy and bronchoalveolar lavage (BAL). However, the relative invasiveness of this technique has limited its use as a practical tool for screening programs, evaluating exposure levels, and conducting repeated follow-up tests in large populations. The ongoing search for noninvasive techniques has led to the development of methods such as analyzing exhaled breath condensate (EBC) and examining cells, mediators, and particulate matter in samples of induced sputum (IS).

In recent years, our research on induced sputum (IS) and exhaled breath condensate (EBC) has focused on occupational and environmental exposure, specifically using micro-sized airborne particles (Dipa 2000 Analyzer Donners Technology). We have studied several populations, including: (a) workers exposed to hazardous dust in Israel [9, 10]; (b) firefighters exposed to World Trade Center dust in the USA [11]; and (c) asthmatic children in the Tel-Aviv area [12]. We have also conducted ongoing complementary measurements of nanoparticulate matter in IS samples using NanoSight technology (NanoSight LTD, London, UK) [13, 14].

In the present study, we use exhaled breath condensate (EBC), fractional exhaled nitric oxide (FeNO), and metabolome measurement in saliva to biomonitor the Haifa population for exposure to ultrafine particles as a surrogate to induced sputum. During the COVID-19 pandemic, sputum induction was classified as an aerosol-generating procedure (AGP), increasing the risk of pathogen exposure and infection [15].

EBC has long been used to monitor toxic effects [16], and FeNO is a simple, safe, and noninvasive method for detecting airway inflammation [17]. Saliva, also known as oral fluid, is a natural filtrate of blood containing small molecules, metals, proteins, and DNA, making it valuable for omics studies such as Epigenome-Wide Association Studies (EWAS). Due to its “stress-free” collection process, saliva was shown to be useful to asses’ biomarkers [18]. Several reviews were published recently [19, 20] summarizing the role of saliva in biomonitoring.

This study is the first to bio monitor the toxic effects of ultrafine particles in Haifa Bay, a heavily industrialized area where numerous factories operate within a confined space. The resulting accumulation of airborne toxins has created unusually high pollution levels, posing potential risks to the health of the local population.

## Methods

### Study population

Fifty healthy medical staff members working in Haifa Hospital and 49 working in Tel Aviv Medical Center were recruited. They were checked at baseline and monitored after 1 year. All participants completed a questionnaire validated by the Epidemiology and Preventive Medicine Department, School of Public Health. Gray Faculty of Medicine and Health Sciences Tel Aviv University.

Some of the data presented in this study were previously included in the final report submitted to the Israeli Ministry of Environmental Protection under Grant No. 180-1-1 The report was submitted as part of the grant requirements and was presented to the Ministry on 25 September 2022 [21]. The report was not published in a peer-reviewed scientific journal.

### Pulmonary function tests (PFT)

These measurements were performed according to standard protocols of the ARS/ERS guidelines and by means of Koko Legend Spirometers (nSpire Health Inc.) [22].

### Exhaled breath condensate (EBC) collection

Exhaled breath was condensed by a TURBO-DECCS condenser (Medivac, Parma, Italy). The condenser has a refrigerating system (TURBO) that thermostatically controls the working temperature, and a disposable respiratory system (DECCS) that consists of a mouthpiece connected to a one-way aspiration valve and an EBC collection test tube at the end. Subjects were asked to perform normal tidal breathing into the mouthpiece for 10 min at an initial condenser temperature of −4 °C to collect 1–2 mL samples which reflect the composition of the airway lining fluid of the lungs. The EBC samples were then analyzed with Nanosight and XRF as described below.

### Saliva collection and processing

Subjects were asked to refrain from eating and drinking 1 h before the session. They were then asked to rinse their mouths with water 5 min before spontaneous saliva was collected in a sterile tube placed on ice. The saliva samples were centrifuged at 4 °C, 13,000 rpm for 30 min. The supernatants were removed and stored at −20 °C and later analyzed for chemical composition as described below. The cell fraction of the saliva samples was suspended with complete cell medium and cytospins were prepared. A total of 200 cells were differentially counted on Giemsa-stained cytopreps with a light microscope.

### Fractional exhaled nitric oxide (FeNO) measurement

FeNO is measured using NIOX VERO (Circassia, UK), in which subjects breathe into the device and the FeNO result is returned in ppb. Values lower than 25 ppb are considered normal and higher indicate inflammation of airways.

Micro particle size distribution (PSD): The size distribution of the particles (particulate matter, PM) was assessed from the rich cell fraction of the processed plugs in IS samples or from the saliva samples by means of a Dipa 2000 Analyzer (Donners Technology, Israel). Briefly, 3 drops of sputum or saliva cells or supernatant suspensions were introduced into a quartz cuvette that contained 3 mL of double distilled water and that were stirred during the analysis. A helium-neon laser beam crossed the particles in suspension, and the signal was registered by a photodiode placed directly behind the suspension. PSD included the following parameters: PM_2.5_PM_5_, PM_10_.

Nano-sized particle measurement: The size of the ultrafine particles (UFP, PM0.1: with diameters of 0.1 μm and smaller) was assessed in the EBC samples by means of Nanosight NS300 (Malvern Panalytical, UK) and the Nanoparticle Tracking Analysis software (NTA, version 3.4, NanoSight Ltd., Salisbury, UK). Approximately 250 µL of EBC samples were inserted into the sample chamber.

The NTA software then utilized the properties of both light scattering and Brownian motion to obtain the size distribution and concentration measurement of the particles in the sample. A 450 nm laser beam was passed through the sample chamber, and the particles’ scatter light was visualized via a ×20 magnification microscope and a camera. The camera operates at 30 frames per second, capturing a video file of the particles moving under Brownian motion (i.e., the smaller the particle, the faster and further it moves). The software tracks many particles individually and calculates their hydrodynamic diameters by means of the Stokes-Einstein equation. Each sample was recorded three times at different positions for average calculation. The parameters that were obtained are: mean particle size (nm); D10, the diameter of 10% of total UFP (nm); D50, the diameter of 50% of total UFP (nm); D90, the diameter of 90% of total UFP (nm); particles concentration (10^8^ particles/mL).

X-ray Fluorescence (XRF) The mineral content of the EBC samples was analyzed with a Thermo Scientific Niton XL3t GOLDD+® XRF analyzer.

Each EBC sample was scanned thrice by XRF, and an average was calculated. Briefly, the instrument was fitted with an X-ray tube with an Ag anode target excitation source (operating at voltages up to 50 kV and at beam currents up to 200 μA), and a geometrically optimized large area drift detector. A helium purge technique was employed for enhanced light element analysis. A charged coupled device camera stored sample images, and the data were transferred via Thermo Scientific Niton data transfer PC software (Thermo Niton Analyzers LLC, version NDT_REL_8.2.1). Calibration was performed each morning before measurements (using control samples: 180-706pp USGS SdAr-M2; NIST 2709a PP 180-649) according to the manufacturer’s instructions.

A total of 200 µL of EBC were placed in the center of a 4 μm polypropylene film over a 3 mm small-spot collimator above the detector. Each measurement took 240 s, and spectra up to 40 keV were quantified with a factory-installed algorithm (fundamental parameters calibration) for a “mining” mode that yielded elemental concentrations in parts per million (ppm, μg/mL) with an error of 2*σ* or 95% confidence.

### Chemical composition measurement

Saliva supernatant chemical composition measurements were performed with the Avdia 2,400 Siemens clinical chemistry system (Siemens Healthineers) in the Biochemistry Laboratory of Tel Aviv Sourasky Medical Center. The parameters that were analyzed are: BUN, Blood Urea Nitrogen (mg/dL using Nirtogen Urease). K, Potassium (mmol/L). CL, Chloride (mmol/L). CA, Calcium (mg/dL). Phos, Phosphorus (mg/dL). LDH, Lactate Dehydrogenase (U/L using Lactate Dehidrogenase).

Statistical analyses were performed using the SPSS® statistics software, version 27.0 for Windows (IBM® corporation).

Results are given in mean ± standard deviation (SD), unless indicated otherwise. Differences between continuous parameters were compared by the *t*-test, and differences between categorical parameters were compared by the *x* ^*2*^ *test*. The *Mann-Whitney U non-parametric test* was used to calculate the differences between small cohorts. Differences between paired observations were calculated with a *paired t-test* or the *Wilcoxon signed rank test*. Pearson correlation coefficients (*r*) were used to correlate between clinical and functional parameters and particles size and other characteristics in biological samples. A p value (p) below 0.05 was considered statistically significant.

LCA is used to detect latent (or unobserved) heterogeneity in samples. The assumption underlying LCA is that membership in unobserved classes can cause or explain patterns of scores across survey questions, assessment indicators, or scales B [23]. We ran latent class analysis (LCA) to identify the number of homogenous subgroups according to selected measurements within the overall sample. A 1 to 3 classes were constructed, and we used three criteria to determine which solution was the best.

The first criterion was whether there were enough participants in each latent class to allow for comparison between classes such that if a class had fewer than 17 participants (20% of the sample), a solution with fewer latent classes was analyzed.

The second criterion was whether the class model solutions is theoretically relevant.

The third criterion was the best fitting model. Better model fit was suggested by a lower Akaike information criterion (AIC) a lower Bayesian information criterion (BIC) a significant Lo-Mendell-Rubin likelihood ratio test, suggesting the more complex model (i.e., model with more classes) fits the data better than the model with fewer classes [24–27]; and entropy, the estimate of certainty of classification (ranging from 0 to 1).

Indicators of the LCA were the following measurements: FEV_1_, FeNO, UFP mean size, BUN, LDH, pH, Cadmium, Palladium, Molybdenum, Niobium and Zirconium.

Once the ideal number of classes was determined, individuals were assigned to their most likely class and were compared between the classes on demographical, clinical and biological variables using univariate analysis.

All analyses related to class formation were conducted using Mplus v8.3 (Muthen & Muthen).

## Results

No differences were observed in demographic characteristics, smoking habits, or functional parameters between the medical staff in Tel Aviv and Haifa (Tables 1,2). However, questionnaire data revealed significantly higher levels of clinical parameters, including atopy (p < 0.001) and the presence of sputum and cough during illness (p < 0.05)—among the Haifa medical staff compared with their counterparts in Tel Aviv (Table 3).

**TABLE 1:** Comparison between demographic data of the Tel Aviv and Haifa medical staffs [Israel, 2019-2024].

​	1st session		2nd session	
Demographic data	TLV Medical staff (N = 49)	Haifa Medical staff (N = 50)	TLV Medical staff (N = 43)	Haifa Medical staff (N = 43)
Age, years	37.92 ± 8.92	40.98 ± 7.11	37.98 ± 9.19	41.21 ± 6.6
Male, *n* (%)	13 (26.5)	17 (34)	11 (25.6)	15 (34.9)
Height, cm	165.52 ± 8.13	167.64 ± 10.41	165.16 ± 8.14	167.7 ± 10.88
Weight, kg	68.56 ± 14.2	73.18 ± 18.3	68.07 ± 14.6	73.93 ± 18.8
Smoking, *n* (%)				
Active	3 (6.1)	1 (2)	2 (4.7)	1 (2.3)
Passive	12 (24.5)	15 (30)	9 (20.9)	14 (32.6)

The p value was calculated with an independent t-test for continuous variables and the *X* ^2^ test for categorical variables and found to be non-significant in all comparisons. Values are given.

**TABLE 2:** Comparison of the pulmonary function test results and fractional exhaled nitric oxide data between the Tel Aviv and Haifa medical staffs [Israel, 2019-2024].

​		1st session		2nd session	
Pulmonary function tests		TLV Medical staff (N = 49)	Haifa Medical staff (N = 50)	TLV Medical staff (N = 43)	Haifa Medical staff (N = 43)
PFT	FEV_1_%	92.87 ± 17.55	97.20 ± 10.58	92.49 ± 12.53	96.65 ± 11.12
	FVC%	95.97 ± 12.57	97.94 ± 11.19	92.98 ± 12.75	97.09 ± 11.43
	FEV_1_/FVC%	99.00 ± 6.66	99.20 ± 8.16	99.23 ± 6.36	99.40 ± 8.39
	FEF_25-75_%	96.62 ± 22.74	99.64 ± 28.59	92.56 ± 22.3	98.81 ± 29.9
FeNO	FeNO, ppb	16.92 ± 10.5	19.7 ± 20	14.41 ± 12.7	15.6 ± 14.5

Results are given as mean ± SD., The p value was calculated with an independent t-test, and all values were non-significant.

FEV_1_, forced expiratory volume in one second, percent of predictive values. FVC, forced vital capacity, percent of predictive values. FEF_25-75_, forced expiratory flow at 25%–75% of FVC, percent of predictive values. ppb, parts per billion.

mean ± standard deviation.

**TABLE 3:** Comparison between questionnaire data of the Tel Aviv and Haifa medical staffs [Israel, 2019-2024].

Questionnaire data	TLV Medical Staff (N = 49)	Haifa Medical Staff (N = 50)
Indoor parameters		
Home with balcony or yard	31 (63.3)	29 (58)
Home facing street	26 (53.1)	31 (62)
Frequent barbeque	7 (14.3)	14 (28.6)
House heating with fireplace	2 (4.1)	1 (2)
House with moisture or mold	13 (26.5)	14 (28.6)
Home furnishings (carpets, curtains, etc.)	25 (56.8)	10 (24.4)*
Pets at home	16 (34)	19 (38)
Outdoor parameters		
Routine outdoor exercise	30 (61.2)	24 (48)
Neighborhood characteristics		
Traffic	30 (61.2)	21 (42)
Noise	25 (51)	18 (36)
Garbage	14 (28.6)	10 (20)
Unpleasant odors	6 (12.2)	8 (16)
Smoke	4 (8.2)	5 (10)
Exposure during military service	7 (14.3)	6 (12)
Clinical parameters		
Cough during sickness	4 (8.2)	13 (26)*
Sputum during sickness	9 (18.4)	23 (46)*
Sensitivity to odors	4 (8.2)	3 (6)
History of atopy	34 (69.4)	11(22) **

^#^ Responses to the questionnaire in the first session were validated during the second session.

Results represent the number of ‘yes’ answers, n (%). The p value was calculated with the *X* ^2^ test.

*p < 0.05, **p < 0.001.

There were no significant differences in the PFT results of the Haifa and Tel Aviv medical staffs. (Data not shown here). The analysis of EBC samples is shown in Table 4. Significant differences were found in EBC UFP size between the medical staff. The mean size was larger in Haifa (231.75 ± 60.19 nm) than in Tel Aviv (166.99 ± 60.22 nm) in the 1st session (p < 0.001) but not in the second session. In addition, the concentration was significantly lower in Haifa in the 2nd session (2.93 ± 1.98 10^8^ particles/mL vs. 5.06 ± 6.02 10^8^ particles/mL in Tel Aviv, p < 0.05).

**TABLE 4:** Comparison between ultrafine particles, X-ray fluorescence and pH measured in exhaled breath condensate of Tel Aviv and Haifa medical staffs [Israel, 2019-2024].

UFP, XRF, pH		1st session		2nd session	
​		TLV Medical staff (N = 49)	Haifa Medical staff (N = 50)	TLV Medical staff (N = 43)	Haifa Medical staff (N = 43)
UFP	Mean size, nm	166.99 ± 60.22	231.75 ± 60.19**	184.38 ± 36.1	181.57 ± 28.81
	D10, nm	88.8 ± 30.63	123.57 ± 36.76**	102.60 ± 18.5	94.18 ± 16.44*
	D50, nm	139.63 ± 57.49	207.5 ± 63.71**	163.28 ± 36.08	160.1 ± 27.27
	D90, nm	281.35 ± 102.27	371.63 ± 92.17**	297.28 ± 63.6	301.39 ± 65.08
	Concentration 10^8^ particles/mL	5.06 ± 4.86	5.44 ± 3.48	5.06 ± 6.02	2.93 ± 1.98*
XRF	Cadmium	27.1 ± 1.9	25.5 ± 1.1**	25.13 ± 0.73	25.42 ± 0.72
	Palladium	10.3 ± 0.63	9.99 ± 0.64*	10.13 ± 0.41	10.12 ± 0.4
	Silver	6.64 ± 1.1	6.4 ± 0.42	6.39 ± 0.4	6.47 ± 0.54
	Molybdenum	18.67 ± 3.5	21.9 ± 2.5**	22.44 ± 1.1	22.56 ± 0.82
	Niobium	21.3 ± 2	22.74 ± 1.63**	22.86 ± 1.07	22.92 ± 0.88
	Zirconium	17.8 ± 0.3	19.05 ± 1.3**	19.13 ± 0.93	19.20 ± 0.78
	Strontium	5.2 ± 0.5	5.3 ± 0.7	5.16 ± 0.46	5.20 ± 0.38
	Tungsten	81.5 ± 17.5	82.4 ± 40.8	81.34 ± 18.5	77.73 ± 13.4
pH	Before DA	7.24 ± 0.17	7.21 ± 0.16	7.18 ± 0.1	7.15 ± 0.06
	After DA	7.71 ± 0.27	7.65 ± 0.27	7.79 ± 0.25	7.72 ± 0.2

Results are given in mean ± SD., The p value was calculated with an independent t-test.

*p < 0.05, **p < 0.001.

D10, the diameter of 10% of total UFP; D50, the diameter of 50% of total UFP; D90, the diameter of 90% of total UFP. UPF, ultrafine particles; DA, de-aeration.

There were also significant differences in the mineral content (XRF analysis) between the two groups only during the first session. The levels of the metal’s molybdenum, niobium and zirconium were significantly higher for the Haifa medical staff (p = 0.001 vs. Tel Aviv medical staff), while the cadmium and palladium levels were significantly lower for the Tel Aviv medical staff (p < 0.001 and <0.05, respectively, vs. Haifa medical staff). There were no comparable differences during the second session. No differences were found in the EBC pH levels between the two groups in both sessions.

The analysis of saliva samples is shown in Table 5. Differential cell count (DCC) analyses were performed in the saliva and there were no significant group differences in any of the DCC parameters.

**TABLE 5:** Comparison between saliva data of the Tel Aviv and Haifa Medical Staffs First and Second Sessions. [Israel, 2019-2024].

DCC, PM, chemistry		1st session		2nd session	
​		TLV Medical staff (N = 49)	Haifa Medical staff (N = 50)	TLV Medical staff (N = 43)	Haifa Medical staff (N = 43)
DCC^#^	%Epithelial cells	81.6 ± 15.9	79.8 ± 13.3	73 ± 22.6	79.9 ± 14.9
	%Neutrophils	18.95 ± 16.3	18.2 ± 12.6	25 ± 21.6	21.7 ± 13.9
	%Lymphocytes	1.91 ± 1.8	2.32 ± 1.5	4.12 ± 3.7	2.46 ± 2.3
PM (Saliva cell fraction)	<2.5 μm %	86.13 ± 9.5	85.45 ± 10.1	82.65 ± 11.17	82.15 ± 10.3
	<5 μm %	93.34 ± 5.8	93.2 ± 6.3	91.09 ± 8	91.03 ± 7.33
	<10 μm %	97.7 ± 2.4	97.62 ± 2.7	96.43 ± 3.93	96.39 ± 3.31
	Size, μm	1.92 ± 0.7	1.98 ± 0.9	2.26 ± 1.1	2.34 ± 0.98
PM + (Saliva supernatant)	<2.5 μm %	80.88 ± 13.9	77.05 ± 14.6	52.60 ± 22.3	51.33 ± 22.12
	<5 μm %	90.6 ± 9.8	90.57 ± 8.5	67.37 ± 20.7	68.33 ± 18.5
	<10 μm %	96.66 ± 4.7	97.6 ± 3.8	81.40 ± 16.2	83.98 ± 13.1
	Size, μm	2.34 ± 1.4	2.32 ± 1.1	6.26 ± 3.9	6.07 ± 3.5
Chemistry (saliva supernatant)	BUN, mg/dL	13.1 ± 4	15.5 ± 5.4*	11.76 ± 3.6	15.65 ± 7.9*
	K, mmol/L	19.2 ± 3.5	19.9 ± 4.7	19.60 ± 3.3	21.22 ± 5.5
	CL, mmol/L	21.0 ± 4.5	23.4 ± 7.2	22.32 ± 6.8	26.98 ± 9.7*
	CA, mg/dL	4.2 ± 1.5	5.1 ± 0.9**	4.80 ± 0.9	5.12 ± 1.08
	PHOS, mg/dL	16.3 ± 3.9	16.9 ± 5.8	16.78 ± 4.4	17.77 ± 6.86
	LDH, U/L	412.1 ± 420	606.6 ± 609*	310.44 ± 195.6	616.02 ± 537.6**

Results are given in mean ± SD., The p value was calculated with an independent t-test.

*p < 0.05, **p < 0.001.

DCC, differential cell count; PM, particulate matter; BUN, blood urea nitrogen; K, potassium; CL, chloride; CA, calcium; Phos, phosphorus; LDH, lactate dehydrogenase.

^#^DCC: 1^st^ session: 0.5%–3% macrophages were counted in 12 samples; 2% eosinophils were counted in 2 samples. 2^nd^ session: 1%–4% macrophages were counted in 8 samples; 1% eosinophils were counted in 2 samples.

+% accumulation of particles in different sizes (< 2.5un; < 5um; < 10um).

The biochemical analysis of the saliva samples showed higher levels in all parameters tested in both Tel Aviv and Haifa during the second session, but the increase in both sessions was significant only in blood urea nitrogen (BUN) and lactate dehydrogenase (LDH). Table 6 displays the negative significant association between the accumulation of particles <2.5 µm, <5 µm and <10 µm in the saliva supernatant and a functional parameter (FVC), indicating that the greater the accumulation of particles, the lower the functional parameters. Testing the association of particle accumulation with functional parameters (FVC and FEV_1_) in Tel Aviv and Haifa groups separately indicates that some of the associations lose their significance in the TLV group but not in the Haifa group.

**TABLE 6:** Pearson correlation coefficient (r) [Israel, 2019-2024].

Correlations	TLV	Haifa	TLV	Haifa	TLV	Haifa
FEV1%#	PM< 2.5 µm		PM<5 µm		NS	
r	NS	-0.301*	NS	0.383*	​	
FEV1%+	EBC pH after de-aeration					
​	TLV	Haifa	​	​	​	​
r	NS	0.289*	​	​	​	​
FVC%#	PM< 2.5 µm		PM<5 µm		PM<10 µm	
​	TLV	Haifa	TLV	Haifa	TLV	Haifa
r	NS	-0.388*	0.389*	-0.526*	0.421	-0.401*
FVC%+	EBC pH after de-aeration					
​	TLV	Haifa	​			
r	NS	0.457*	​			
FeNO (ppb)	D10 nm		D50 nm			
​	TLV	Haifa	TLV		Haifa	
r	NS	0.299*	NS		0.320*	

*p value < 0.05 NS, not significant.

FEV_1_, forced expiratory volume in one second, percent of predictive values.

FVC, forced vital capacity, percent of predictive values.

#Pearson Correlation between functional parameters and PM, in the saliva supernatant.

+pH after De-aeration in EBC, between and FeNO, the Tel Aviv and Haifa groups.

ppb, parts per billion; nm, nanometer; D10, the diameter of 10% of total UFP; D50, the diameter of 50% of total UFP; D90, the diameter of 90% of total UFP.

The acidification of airways was measured by pH in EBC. There was a positive significant association of PH with functional parameters (FVC and FEV_1_) in the Haifa group, indicating that a low pH is associated with low functional parameters.

Inflammation in airways was measured by FeNO, and positive significant associations between FeNO and UFP size distribution and between FeNO and mean size were revealed, indicating that UFP of all sizes induce elevated values of FeNO.

Table 7 presents the fit indicators of the LCA procedure. The first criterion for a minimum of 20 participants in class ruled out the 3-Class solution.

**TABLE 7:** Latent Class Analysis Model estimation fit indices, N = 99 [Israel, 2019-2024].

Minimal no. of participants in each class	Entropy (0–1)	LMRT (p<0.05)	BIC↓	AIC↓	Class
99	​	​	6,715.605	6,653.322	1
34	1.00	0.0271	6,316.983	6,220.964	2
5	0.996	0.5754	6,304.223	6,174.467	3

LMRT, Lo-Mendell-Rubin Test; AIC, akaike information criterion; BIC, bayesian information criterion.

2 Class solution has a lower AIC and BIC than the 1 Class solution and a significant Lo-Mendell-Rubin likelihood ratio test, therefore we identified the 2 Class model as optimal. The first class consisted of 34 subjects and the second class consisted of 65 subjects (Table 8).

**TABLE 8:** Latent class analysis of the medical staffs–further analysis [Israel, 2019-2024].

Further	Analysis		Cluster 1 (N = 34)	Cluster 2 (N = 65)	​
Study group	TLV		30 (88.2%)	19 (29.2%)*	​
	Haifa		4 (11.8%)	46 (70.8%)	​
Sex	Male		8 (23.5%)	22 (33.8%)	​
	Female		26 (76.5%)	43 (66.2%)	​
BMI			23.9 (4.66)	26.1 (4.65)*	​
EBC	UFP mean size, nm		151 (64.6)	225 (55.2)**	​
	UFP D10, nm		83.3 (36.9)	118 (32.8)**	​
	UFP D50, nm		125 (60.3)	199 (59.9)**	​
	UFP D90, nm		252 (106)	366 (85.1)**	​
	UFP concentration, 10^8^/mL		3.56 (2.47)	6.13 (4.65)**	​
	XRF Cd, ppm		28.2 (1.41)	25.3 (0.73)**	​
	XRF Pd, ppm		10.3 (0.97)	10.1 (0.40)	​
	XRF Ag, ppm		8.63 (1.70)	6.39 (0.43)	​
	XRF Mo, ppm		15.8 (0.99)	22.6 (1.19)**	​
	XRF Nb, ppm		19.8 (0.93)	23.2 (1.25)**	​
	XRF Zr, ppm		16.5 (0.79)	19.4 (1.08)**	​
	XRF sr, ppm		5.03 (0.44)	5.33 (0.66)*	​
	XRF W, ppm		77.8 (17.3)	83.6 (36.6)	​
	PH before de-aeration		7.24 (0.19)	7.21 (0.16)	​
	PH after de-aeration		7.75 (0.28)	7.64 (0.26)*	​
Saliva	BUN, mg/dL		13.2 (3.90)	14.8 (5.30)	​
	CA, mg/dL		3.75 (1.50)	5.04 (0.92)*	​
	LDH, U/L		361 (488)	581 (541)	​
	LDH<=330 U/L		20 (69.0%)	23 (35.9%)*	​
	LDH>330 U/L		9 (31.0%)	41 (64.1%)	
	Saliva %Neutrophils		20.2 (18.3)	17.6 (12.1)	​
	Saliva %lymphocytes		1.29 (0.95)	2.43 (1.65)*	​
	Saliva %epithelial cells		79.4 (18.8)	81.7 (11.6)	​
Questionnaire	Atopic No		13 (38.2%)	41(63.1%)*	​
	​	Yes	21 (61.8%)	24 (36/9%)	
	Cough during sickness		​	​	​
	​	No	13 (38.2%)	50 (76.9%)	0.061
	​	Yes	21 (61.8%)	15 (23.1%)	
	Sputum during sickness		​	​	​
	​	No	26 (76.5%)	41 (63.1%)	​
	​	Yes	8 (23.5%)	24 (36.9%)	

*p < 0.05, **p < 0.001; nm, nanometer; ppm, part per million.

We analyzed the association between the clusters and the demographic and clinical parameters (Table 8), and found that cluster 2 was characterized as having:

- Greater proportion of the Haifa population.
- Higher BMI levels.
- Higher UFP concentrations in EBC.
- Higher levels of calcium and LDH (above 330U/L) in saliva.
- Higher percentage of lymphocytes in saliva.
- More atopic symptoms.

This cluster included more Haifa individuals with high concentrations of UFP in EBC. The biochemical analysis of the saliva showed high concentrations of LDH (as a marker of degradation). The inflammatory effect of the higher concentration of UFP was depicted by symptoms of atopy and high percentages of lymphocytes in saliva.

## Discussion

Biomonitoring urban pollution involves using biological systems—such as plants, animals, or microorganisms—to assess the level and impact of pollution in urban areas. We present here the use of different biological samples to monitor pollution in two big cities in Israel.

The population studies in Tel Aviv and Haifa displayed the same PFT values and showed no differences between them when compared individually in the first and second session. These results confirmed those of our earlier studies on other worker populations compared to normal populations [28]. PFT values are not sufficiently sensitive to serve as a tool for biological monitoring.

We therefore collected saliva and EBC as less invasive alternatives to induced sputum.

Saliva, also known as oral fluid, is a natural filtrate of blood that contains various omics features, including small molecules, metals, proteins, and DNA. It offers numerous advantages over other biofluids, as it can be collected safely and noninvasively with minimal training. Additionally, saliva is rich in biological information [18]. The multiple functions provided by Saliva are essential for proper protection and functioning of the body as well as for general health. With the spread of the COVID pandemic, the saliva-based molecular tests have shown a similar sensitivity and specificity compared to nasopharyngeal tests for SARS-CoV-2 [29, 30]. The use of saliva in other pathological conditions, such cancer [31], autoimmune disease [32] and Alzheimer disease [33] have also been reported.

In our current investigation, we demonstrated the validity of our methods using samples of saliva and EBC to measure the mean size of particulate matter in the airways. It is known that inhaled particles distribute in the airways according to their size and become smaller in the deeper sites in the bronchial tree. They deposit efficiently in the human lung and can translocate to the systemic circulation and deposit into various organs [34–36]. It emerged that the mean size of particles in saliva was significantly larger than those measured in EBC (data not shown here) since the saliva sample recovered from an upper airway compartment. These results agree with those shown by us in a previous study where we showed that particles recovered from bronchioalveolar lavage are smaller than those recovered from induced sputum [37]. The comparison of these very significant changes in the size of UFP particles between the two populations at two periods of time may indicate that this parameter can be very sensitive to some unpredictable atmospheric changes like those resulting from lockdown that can act as a confounder. This was demonstrated in a very recent observational study that showed that reduced levels of air pollution during the COVID-19 lockdown in Israel were reflected in increased levels of UFP airway contents [38].

Interestingly, the large size of particles in Haifa and the high concentration of UFP particles occurred in parallel with high levels of metals during the first session. During the second sessions the size decreased, and the level of concentration lowered by almost one-half (5.06 ± 6.02 vs. 2.93 ± 1.98). This phenomenon can be explained by the fact that metals attached to the surfaces of particles exhibit enhanced properties due to their small dimensions and high surface-to-volume ratio [39].

The results of all the biochemical parameters were consistently higher in Haifa compared to Tel Aviv in both the first and second session, but significant for two of them (LDH and BUN). We focused particularly on LDH levels, as this metabolite has been identified as an indicator of cellular integrity disturbances and a marker of lung and pulmonary endothelial cell injury [40]. Blood urea nitrogen (BUN) is a serum byproduct of protein metabolism. It is one of the oldest prognostic biomarkers of heart failure. Urea is formed by the liver and carried by the blood to the kidneys for excretion and may be used as a marker for predicting renal disease.

Accumulation of PM in saliva samples (PM 2.5 µm, PM 5 µm, and PM 10 µm) were negatively associated with PFT parameters (FVC and FEV1), indicating that the higher the accumulation of this fraction of PM the worse will be the PFT parameters. This correlation remained significant only in the Haifa population when the two urban populations were compared. The fact that inhaled particles correlated with worse PFT results had already been reported in elderly [41] and in a pediatric population [42]. While those studies used measurements in the environment PM, however, to the best of our knowledge ours is the first report on PM findings in Saliva.

A negative association was found between pH and PFT, demonstrating that acidification of the airways (i.e., lower pH) was correlated to worse PFT results supporting our earlier findings [28].

FeNO was used in this study as a surrogate for eosinophils in IS and its concentration was shown to be positively associated to the accumulation of UFP. This was significant only in the Haifa study population. These results may indicate that small particle accumulation in the airways as measured in EBC caused high inflammatory values of FeNO in the Haifa population.

Epidemiological and toxicological research support a link between air pollution and an increased incidence and/or severity of airway inflammation. FeNO is a simple, safe and noninvasive method to detect airway inflammation. It is also correlated well with eosinophil count and eosinophil cationic protein in induced sputum and serves as a widely used metric for the evaluation and management of airway inflammation [43–45].

In this context toward this end, we performed a latent class analysis (LCA) to identify the number of homogenous subgroups according to selected measurements within the overall sample (detailed in the Statistical Methods section). Once the ideal number of classes had been determined, individuals were assigned to their most likely class and compared between the classes based upon demographic, clinical, and biological variables by means of a univariate analysis.

The results yielded two homogeneous clusters. The measurements chosen as indicators of the LCA were: FEV_1_(the only physiological parameter), FeNO, UFP (very small particles mean size) pH, cadmium, palladium, molybdenum, niobium and zirconium in EB, BUN, LDH in saliva. These markers were selected according to the parameters that had been shown to be with significant differences and sensitive all over the study performed.

Cluster 2 included more Haifa individuals who had high concentrations of UFP in EBC. The biochemical analysis of the saliva showed high concentrations of LDH (as a marker of degradation). The inflammatory effect of the higher concentration of UFP was reflected by symptoms of atopy and high percentages of lymphocytes in saliva.

### Conclusions and limitations

The present study represents an initial attempt to identify a potential profile of biological tests that may, in the future, serve as the basis for a comprehensive model to bio-monitor the health status of the population in Haifa city. Such a model would allow the integration of specific biological parameters that could act as sensitive indicators of early physiological changes and potential health risks associated with environmental exposure. By defining which of these parameters are most reliable and informative, it will be possible to establish a framework for detecting the deleterious effects of environmental factors on the local population and to guide preventive health strategies and public policies.

Despite these promising directions, the current study has several limitations that must be acknowledged. An important limitation is that environmental monitoring data were not collected, as this was not included in the initial design of the research proposal. The absence of direct environmental exposure measurements makes it more difficult to establish clear causal links between environmental pollutants and the biological changes observed. Future studies should therefore aim to expand the sample size, incorporate environmental monitoring of air, soil, and water pollutants. These improvements will strengthen the validity of the findings and enable a more comprehensive understanding of how environmental exposures affect the health of the population in Haifa.
